# Secretome Analysis Using Affinity Proteomics and Immunoassays: A Focus on Tumor Biology

**DOI:** 10.1016/j.mcpro.2024.100830

**Published:** 2024-08-13

**Authors:** Vanessa M. Beutgen, Veronika Shinkevich, Johanna Pörschke, Celina Meena, Anna M. Steitz, Elke Pogge von Strandmann, Johannes Graumann, María Gómez-Serrano

**Affiliations:** 1 Institute of Translational Proteomics, Biochemical/Pharmacological Centre, Philipps University, Marburg, Germany; 2 Core Facility Translational Proteomics, Biochemical/Pharmacological Centre, Philipps University, Marburg, Germany; 3 Institute of Pharmacology, Biochemical/Pharmacological Centre, Philipps University, Marburg, Germany; 4 Institute for Tumor Immunology, Center for Tumor Biology and Immunology, Philipps University, Marburg, Germany; 5 Translational Oncology Group, Center for Tumor Biology and Immunology, Philipps University, Marburg, Germany

**Keywords:** affinity proteomics, antibody arrays, immunoassays, extracellular vesicles, Olink, secretome, SomaScan, RPPA

## Abstract

The study of the cellular secretome using proteomic techniques continues to capture the attention of the research community across a broad range of topics in biomedical research. Due to their untargeted nature, independence from the model system used, historically superior depth of analysis, as well as comparative affordability, mass spectrometry-based approaches traditionally dominate such analyses. More recently, however, affinity-based proteomic assays have massively gained in analytical depth, which together with their high sensitivity, dynamic range coverage as well as high throughput capabilities render them exquisitely suited to secretome analysis. In this review, we revisit the analytical challenges implied by secretomics and provide an overview of affinity-based proteomic platforms currently available for such analyses, using the study of the tumor secretome as an example for basic and translational research.

## Highlights

• Comparison of approaches to secretomic analysis: strengths and opportunities.
• Overview of affinity proteomic approaches, including newly emerging techniques.
• Application to the study of the tumor microenvironment and related biofluids.

## Graphical Abstract

**Figure:**
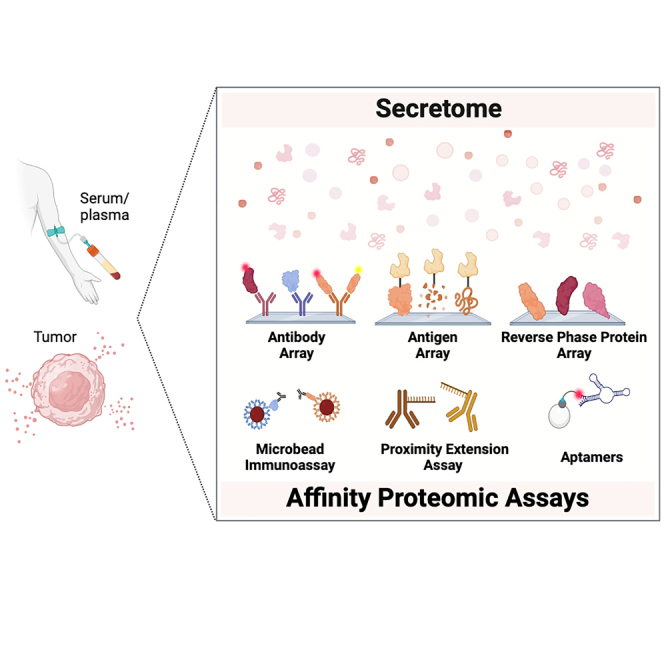

## In Brief

Affinity- and mass spectrometry-based methods represent complementary technologies for proteomic secretome analyses. While mass spectrometry-based methods allow for unbiased analysis but requires extensive sample preparation, affinity-based, high-throughput proteomic platforms cover a broad dynamic range and suit large explorative studies and clinical trials. Certain affinity formats provide enhanced sensitivity for low-abundance proteins, advantageous for assay development while monitoring predefined biomarkers. This review provides an overview over affinity-based technologies for secretome analysis, with an emphasis on cancer as a heavily studied example.

The cellular secretome and its changes, which mirror physiological and pathological processes such as carcinogenesis, are thought to harbor great potential for the identification of process-characteristic molecular features, which in turn are expected to serve in the clinical setting as biomarkers for diagnosis as well as for monitoring of treatment and disease progression. Classical approaches to decipher the proteinaceous secretome in general, and that of tumor cells in particular, have involved the analysis of conditioned medium (CM) from primary cells or established cell lines using mass spectrometry- (MS) based methodology. These approaches commonly imply sample preparative steps including protein enrichment and separation to achieve a deep representation of the cellular secretome from diluted input material. While CM frequently presents with an overall lower protein complexity as compared to body fluids such as blood, urine, cerebrospinal fluid (CSF), or ascites from the peritoneal cavity, its preparation for MS analysis remains challenging.

The search for biomarkers in the secretome commonly follows a three step-process including the following: [1] initial untargeted discovery screening using MS-based methods, proceeding to [2] candidate-specific validation using classical immunoassays such as ELISA, immunohistochemistry or Western blotting (WB), and [3] clinical translation and verification through extended, high throughput analysis of body fluids and/or liquid biopsies. Matrices clinically accessible with minimal invasiveness such as blood, urine, or saliva are of special interest in this context, as they imply the potential for routine use in medical settings.

In this multistep discovery and validation process for biomarkers originating in tissue culture, it is implicit that proteins secreted *in vitro* are expected to share that behavior *in vivo*. However, while providing reduced experimental variance and ease of access, culture-based screens do not necessarily reflect the actual *in vivo* microenvironment, or the influence of a pathological state such as cancer. Direct candidate biomarker screening from patient blood, and in particular proximal fluids collected at a disease site, is thus assumed to yield biomarker candidates with a much-improved likelihood of validation. Modern proteomic screening methods, and especially approaches using affinity-based platforms and immunoassays, render such biomarker screens directly from the terminally targeted analyte feasible. These approaches meet the challenges posed by sample characteristics as well as the large biological variance implied in analyzing patient-derived material. In this review, we provide an overview of the opportunities offered by state-of-the-art affinity-based proteomics methods, both established and novel, for the screening of secretome biomarkers, using the study of the tumor secretome as an exemplary complex setting.

## The Human Secretome

### Definition

**Fig. 1:**
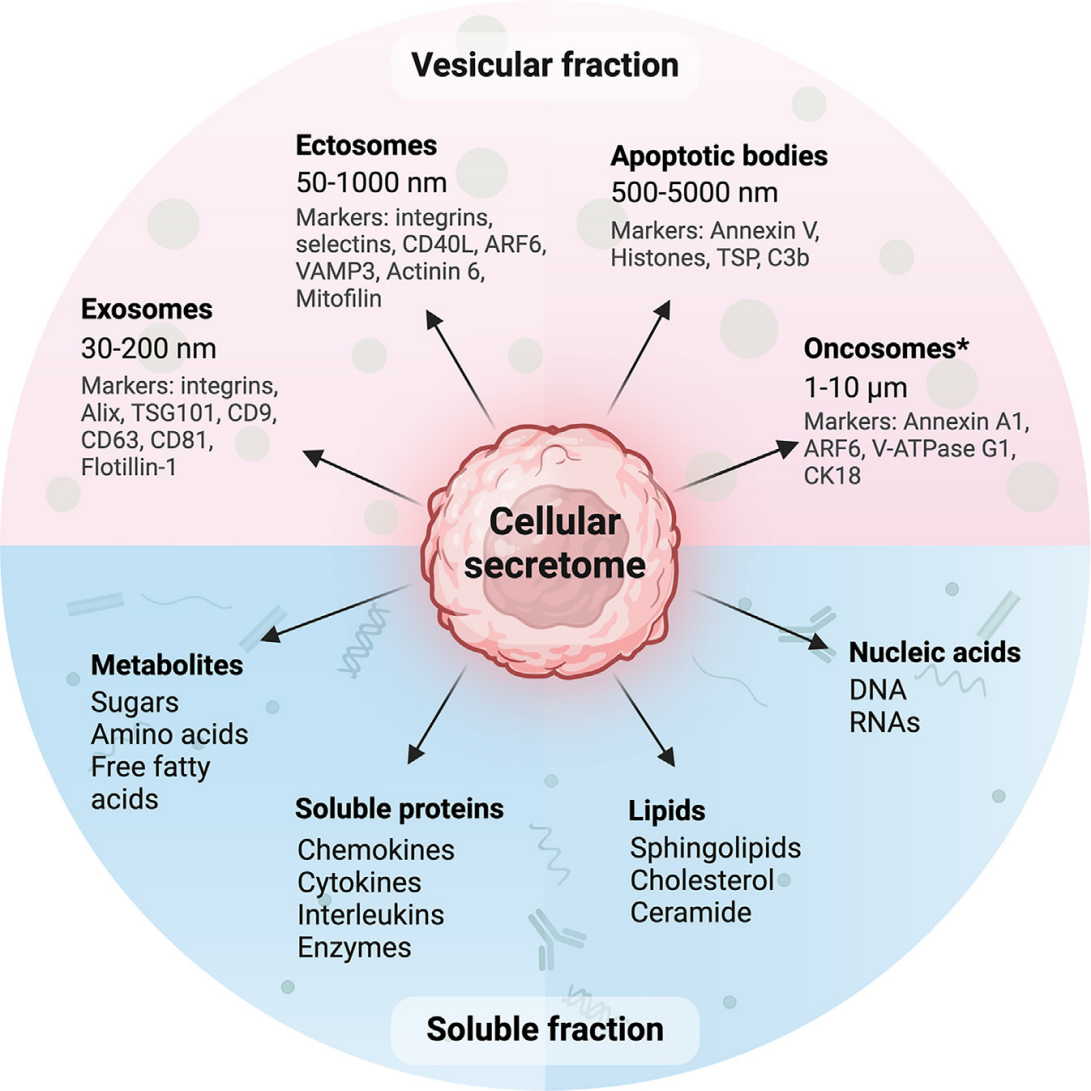
**Components of the cellular secretome.** Two main constituents may be distinguished: a soluble fraction, including metabolites, lipids, nucleic acids, and proteins in soluble forms; and a vesicular fraction. Only major vesicle classes based on their biogenesis routes and size are indicated here, including some well-established markers for their identification. Oncosomes (indicated with ∗) are only relevant for tumor cells. The molecular cargo of vesicles also consists of metabolites, lipids, nucleic acids, as well as proteins. Created with BioRender.

The cellular secretome is commonly considered to include all factors released from a cell. Whether this release must necessarily be active, or if secretion of molecules *via* “passive” processes (*e.g.*, during apoptosis or necrosis) is also comprised, remains a matter of frequent debate. Regardless of the origin of the molecules and whether terminology presuming “secretion” (*i.e.*, active externalization) is entirely appropriate or not, biofluids carry a complex complement of molecules in which both actively and passively released actors may be found, exerting potential global effects on an organismic level and/or acting as biomarkers. Rendering the question of what comprises the secretome even more challenging is the fact that it contains a soluble compartment and a particle-based or vesicle-associated one (Fig. 1), as well as a plethora of biochemical molecule classes (proteins, metabolites, nucleic acids, and lipids) differentially distributed between them. An all-encompassing definition of the secretome is beyond the scope of this review, but given that the majority of “secretome” studies to date focus on its proteinaceous components, this review also does so. The term “secretome” will thus henceforth refer to the cell-derived complement of proteins in a given matrix, irrespective of the subcellular source and compartment they are comprised in.

Alterations of the cellular secretome have been associated with a wide range of disorders, such as cardiovascular disease (1) or obesity (2). Likewise, the study of the secretome is gaining relevance in tumor biology, as it has been demonstrated to significantly differ between cancer and healthy cells, rendering it particularly interesting for biomarker discovery (3, 4, 5).

### Secreted Proteins and the Human Protein Atlas

There are three well-characterized active protein secretion pathways: the classical pathway *via* signal peptides and transport through the Golgi apparatus; the nonclassical, Golgi-independent pathway (“unconventional protein secretion”), and the endosomal pathway (6). While novel strategies such as proximity labeling and CLICK-chemistry (reviewed in (7)), are helping to analytically identify protein substrates of specific secretion mechanisms, the definition of a given protein as “secreted” remains largely based on *in silico* analysis information aggregated from dissimilar data sources (7). One of the most comprehensive resources of secretomic data to date is provided by the Human Protein Atlas (HPA) (Available at: https://www.proteinatlas.org/humanproteome/blood+protein) (8). This project uses data from genome-wide association studies to predict the total number of human secretory proteins (6, 9). Putative secretory proteins are defined as proteins having a signal sequence for translocation to the endoplasmic reticulum (ER) and lacking transmembrane regions. According to its most recent revision (accessed on 26.02.2024), this applies to 2771 proteins of which 780 are categorized as blood secretome proteins. Additionally, the resource compiles results from various efforts to decipher human plasma protein profiles in healthy individuals. Based on MS data, 4066 proteins (secreted and leakage proteins) are thus cataloged and their concentrations estimated based on spectral counts. The HPA further includes curated information on absolute plasma protein concentrations for 438 actively secreted proteins based on different immunoassay studies. It is noteworthy that beyond the leakage proteins included in plasma in the HPA based on MS evidence, the resource likely misses proteins secreted *via* unconventional protein secretion, as these miss a signal peptide (10) and, accordingly, cannot be easily identified by sequence analysis alone. Unconventional protein secretion pathways are categorized into vesicle-independent and -dependent transport routes. It is further noteworthy that organelles involved in the latter, like multivesicular bodies, lysosomes, and autophagosomes are also considered root organelles for the formation of extracellular vesicles (EVs) (11). The mechanisms which control the recruitment and sorting of proteins to EVs are not well-studied at the molecular level, and protein motifs directing the unconventional EV-associated secretion are still not defined (12). Further research and secretome analyses using proteomics will provide insight in the general rules and specific protein motifs involved, and thus improve our understanding of protein secretion through EVs.

### Contribution of EVs to the Human Secretome

EVs were initially considered cellular garbage (13) and, later, interpreted as an additional cellular strategy to discard obsolete proteins (14, 15), in particular in the context of ER stress and the unfolded protein response. It has indeed been shown that both processes directly impact the secretome by decreasing the transcription of secreted proteins and by eliminating misfolded proteins through the ER-associated protein degradation pathway (16), and furthermore, that unfolded protein response is linked to vesicle release (17). Beyond those early hypotheses, EVs have become recognized as integral components of the functional secretome based on their observed vital role in intercellular communication. EVs are broadly defined as nanosized cell-derived particles bounded by a lipid bilayer membrane, unable to replicate on their own (18, 19). They are secreted from virtually all cells and may be found in all body fluids such as plasma, serum, urine, and ascites (20), carrying cargo of large diversity, including proteins, RNA, and lipids. Current efforts to map the entire landscape of EV cargo are reflected in public data repositories like ExoCarta (21) or Vesiclepedia (22), as well as in recent high-throughput studies (23). Several EV classification frameworks have been proposed, but their division into the main subcategories of exosomes (30–150 nm) and ectosomes (100–1000 nm) based on their size, biogenesis, and cargo proteins is the most commonly used, as recently reviewed (24, 25, 26, 27). However, and despite these reports, canonical markers for different EV subtypes remain to be universally accepted (19).

Given their role in cellular communication, it is not surprising that EVs majorly impact the biology and function of cancer cells as well as other cells of the tumor microenvironment (28, 29). Recently, tumor-derived EVs (T-EVs) have attracted particular attention due to cancer type-specific protein cargo, reflecting the state of the tumor cells from which they originate (30, 31). Moreover, concentrations of circulating EVs have been found to correlate with disease severity and prognosis in several cancer entities, rendering them promising targets for liquid biopsy biomarker discovery in cancer research (32, 33, 34, 35). In line with this concept, T-EVs have been found to contain oncoproteins, whose characterization may improve early diagnosis or treatment monitoring in a broad range of malignancies (for an extensive review see (28)). For instance, ADAM8, which is overexpressed in pancreatic ductal adenocarcinoma (PDAC) cells, is found enriched in serum-derived EVs from corresponding patients (36); and, PDAC patients developing liver metastases present with elevated levels of migration inhibitory factor in EVs as compared to those with stagnant tumors (37). Moreover, the classical ovarian cancer (OC) biomarkers CA125 (also known as Mucin-16 or MUC-16), HE4, and C5a were found in serum-derived EVs from epithelial OC patients providing greater diagnostic potential than their detection in serum alone (38).

While biomarker profiling through EVs carries promises, multiple technical aspects complicate their study, starting with the standardization of isolation methods, a key barrier to working with EVs in a clinical setting. Several approaches to isolate EVs are available (reviewed by (39)) but the individual method of isolation strongly impacts the detectable protein profile as none of them produces pure EV fractions and carries characteristic amounts of contamination or EV-associated factors (39). Moreover, EVs comprise a wide range of distinct vesicle classes with varying sizes and cargo, and different isolation methods tend to influence the corresponding composition of the final sample. Results of studies using different approaches must thus be compared with caution. Comprehensive overviews of EV isolation methods and their implications for MS-based proteomics have recently been published (40, 41). EV analyses using affinity-based proteomics remain scarce and the technology platform likely underexplored. Lack of canonical EV markers, heterogeneity of EV subpopulations and relative novelty of affinity approaches and the EV research field itself, may partially explain the limited literature coverage of the topic. Where applicable, representative EV-studies using affinity methods will thus be highlighted in this review.

## Facing Challenges in Secretomics through Affinity Proteomics

The analysis of the secretome entails specific technical challenges dependent on the sample type, collection method, sample handling, and storage, as well as analysis method. A recent review has outlined several challenging aspects in MS-based secretomics, some of which extend to affinity-based proteomics approaches (42). As mentioned above, the sample types most frequently used in secretomic studies comprise CM, blood-derived samples, or liquid biopsies like CSF and ascites. Common features of these samples are high complexity and protein concentrations distributed over a wide dynamic range. Notably, the proteins of interest are often present at very low concentration (as is for example the case for cancer antigens like prostate-specific antigen (PSA), carcinoembryonic antigen (CEA), and CA125 (43)), and are easily obscured by other highly abundant proteins in the sample. Additionally, as discussed above, secretome samples may potentially be “contaminated” by nonsecretory proteins resulting from cell lysis and necrotic or apoptotic events, but also shed membrane proteins, and other cellular debris. This renders it challenging to identify actively secreted proteins and to discover, for example, true tumor-associated proteins (44, 45). For instance, the overlapping secretion profiles between senescence-associated secretory phenotype factors and those originating from tumor cells have posed difficulties in the identification of tumor-specific secreted proteins (46).

Proteomics of CM from cultured cells is a widely used approach to analyze the secretome of specific cell types under various conditions. Cell culture models, however, are suboptimal tools for the characterization of complex mechanisms of pathogenesis *in vivo*. Nevertheless, culture models imply potential for more focused research questions, superior standardization of analysis techniques and replication, as well as avoidance of interindividual variation (*e.g.*, immortalized cell lines). In summary, secretome analysis, also in cell culture models, faces the three major challenges of contamination, concentration, and separation (47).

### Contaminating Proteins in Cell Culture

Culture conditions may influence the outcome of the secretome analysis and introduce biases and contaminants. Cultured cells frequently require serum (*e.g*., fetal bovine serum) and other supplements in the medium, which in turn contain highly abundant proteins such as albumin or immunoglobulins (*e.g.*, IgG). To prevent masking of low-abundant secretory proteins, the medium is generally exchanged for serum-free formulations prior to sample collection, potentially inducing stress responses in cells and compromise cell viability, thereby altering the protein composition of the CM as compared to standard culture. Challenges arising in this context were also recently reviewed by Wu and Krijgsveld (7). To mitigate these disadvantages, MS-based methods frequently apply metabolic or proximity labeling approaches devoid of starvation effects, allowing a selective enrichment of secreted proteins (reviewed in (42)). In affinity-based proteomic technologies, cross-reactivity of human protein-targeting reagents with *e.g.*, bovine serum proteins is an additional challenge. Where culturing of cells in serum-free media prior to analysis is not possible, parallel analysis of control cell culture media as a negative control may be used to determine background levels of such interference to define a specific limit of detection (LOD) for each assay (48). This approach still allows the semiquantitative protein measurement of proteins above the defined threshold from signals of bovine orthologs, but comes at the cost of a reduced overall protein detectability. Additionally, cells may be damaged during sample collection, releasing intracellular, secretome contaminating, proteins into the medium (47). A further problem with this sample type is a frequent high-dilution factor for secreted proteins in a large sample volume and the resulting need for protein enrichment prior to analysis (*i.e.*, trichloroacetic acid precipitation and ultrafiltration), often resulting in poor recovery and potentially reducing the spectrum of detectable proteins. Even though the complexity of CM samples is commonly inferior to that of, for example, plasma samples, conventional methods applied to their analysis also require extensive sample preparation and orthogonal validation. For instance, sample fractionation has been shown to decrease sample complexity (*e.g.* strong anion/cation exchange chromatography, high-pH reversed-phase fractionation, SDS-PAGE, or combinations thereof), and, correspondingly, to increase depth of the analysis (49). Furthermore, MS-based protein identification is often followed by a validation step in the form of immunoassays such as WB or ELISA. Affinity-based proteomics has the advantage of allowing samples to be analyzed without the need to deplete high-abundance proteins. Given that it is based on specific interactions between target proteins and capture agents (*e.g.*, antibodies and aptamers), affinity-based methods demonstrate high specificity and enable accurate identification and quantification of proteins, a common challenge in LC-MS (50). Furthermore, researchers have the option to design custom affinity assays with capture agents for specific targets of interest, largely circumventing the challenges of protein contamination.

### Protein Concentration Range in Clinical Samples

Blood-derived sample material is easily accessible using minimally invasive methods. Serum and plasma contain, however, a plethora of different molecules, rendering them matrices of high complexity. In fact, protein concentrations in these samples show a dynamic range of more than ten orders of magnitude, ranging from concentrations of fg/ml to mg/ml. As a result, 99% of the plasma proteome is composed of just 22 highly abundant proteins (51). In MS-based analyses, low-abundant proteins are often obscured by omnipresent proteins such as albumin or immunoglobulins. Proteins of clinical interest are, however, frequently functionally relevant at very low concentration ranges, either as disease-related protein leakage into the plasma or as inherently low-abundant proteins like hormones, cytokines, or growth factors (52). It should further be emphasized that circulating proteins are not organ-specific and a clear association with a tissue-specific pathological process may prove challenging, as locally released proteins are further diluted when entering the blood flow.

### Avoiding Protein Separation

**Fig. 2:**
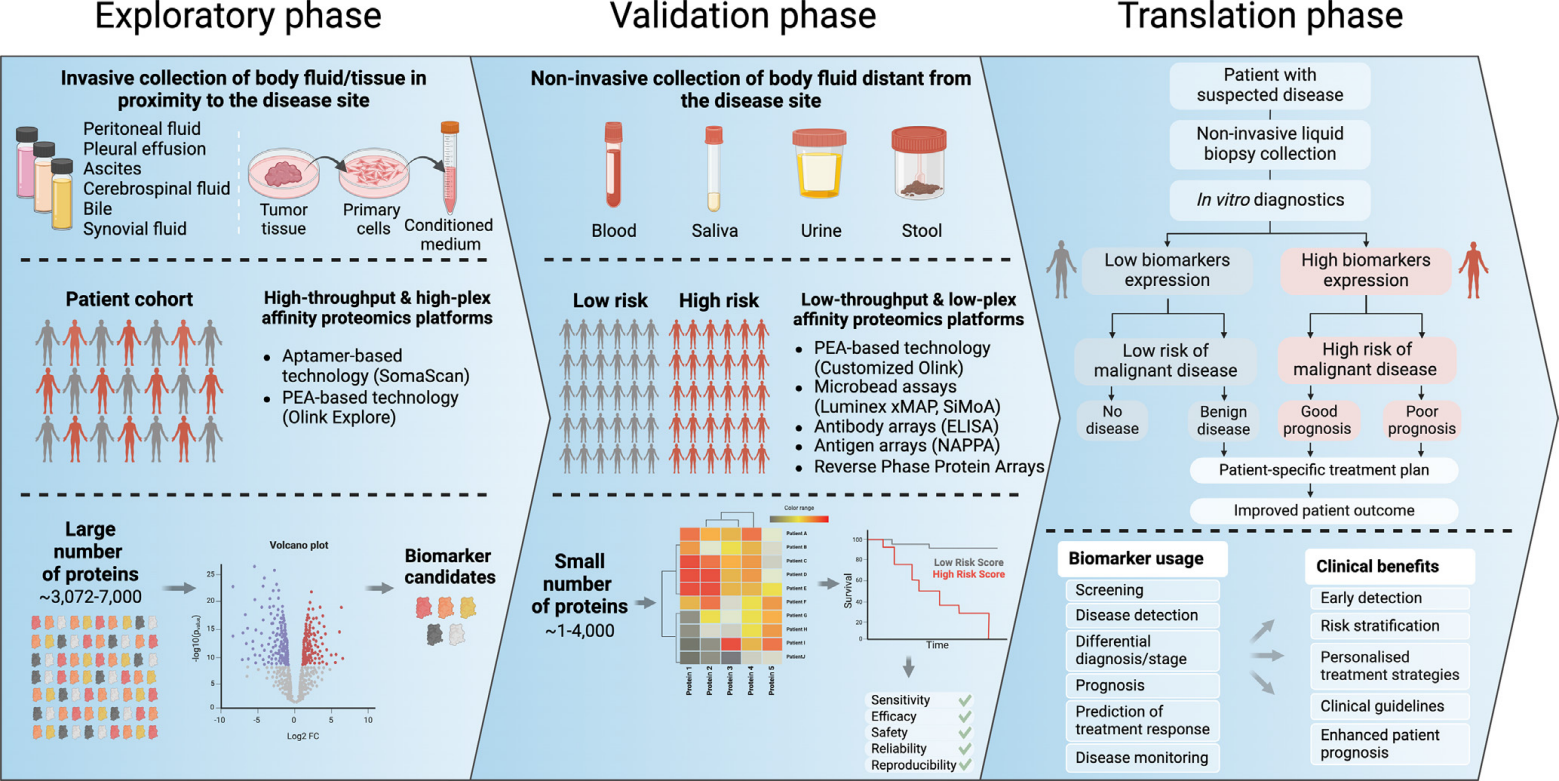
**Schematic biomarker discovery pipeline in cancer research using secretome affinity proteomics approaches.** The exploration phase comprises collecting biomaterials in close proximity to the disease site, including body fluids or conditioned medium generated from primary cells of a tissue biopsy. This step also involves studying a diverse patient cohort and using high-throughput and high-complexity affinity proteomics (AP) platforms. AP analysis enables the simultaneous screening of a large number of proteins, including low-abundant proteins, and allows identification of potential biomarkers from this pool of candidates. The validation phase involves the collection of liquid biopsy specimens at sites remote from the disease focus. In this step, patient cohorts are categorized by risk factor and investigated using low-throughput and low-plex affinity proteomics (AP) assays. Comprehensive evaluation of selected biomarkers includes testing their efficacy, sensitivity, reliability, safety, and reproducibility to obtain an understanding of their performance and suitability for specific clinical applications. In the translational phase, patient stratification is accomplished using *in vitro* diagnostics of non-invasive liquid biopsies, allowing for the assessment of protein expression levels. The utilization of the identified biomarkers serves a variety of clinical applications, bringing meaningful benefits to patients. Created with BioRender.

To increase the detectability of the low-concentration range proteins in serum or plasma, depletion of highly abundant proteins and various forms of sample prefractionation are frequently employed in the context of MS-based analyses. Because every additional step in an analysis pipeline potentially introduces bias, their restriction to a minimum number is desirable. Depletion of highly abundant components may unintentionally codeplete potential protein biomarker candidates, as highlighted for CSF (53). Affinity proteomic approaches circumvent the needs for depletion steps with high dynamic range capabilities, while simultaneously detecting low-abundance proteins in complex samples with high sensitivity. Biofluids used for affinity analyses so far include urine (54), peritoneal fluid (55), ascites (56), CSF (57), aqueous humor (58), synovial fluid (59), saliva (60), and stool (61) but are not conceptually limited to those. Body fluids like urine, saliva, or blood are more easily accessible and may be used for multiple sampling, allowing longitudinal monitoring of potential disease markers (Fig. 2). In contrast, liquid biopsies collected in the proximity of disease foci (*e.g*., aqueous humor or synovial fluid), may deliver more tissue- and organ-specific proteomic profiles. A major proportion of the secreted proteins remains local and may provide insight into the cellular state of immediately surrounding cells. The analysis of the proteomic profile of the tumor microenvironment may, in particular, deliver new insight into the disease state and progression in cancer research. On the other hand, liquid biopsy samples like pleural effusion, peritoneal fluid, or CSF are collected ethically only during curative or debulking surgery and thus accessible only infrequently. Implicitly availability of healthy counterpart control samples is limited. Affinity-based methods commonly require very small sample volumes, making them suitable for limited or high-value samples like those mentioned above. Moreover, customized affinity analyses can be developed to address very specific research questions, again while circumventing problems introduced by protein separation.

### Additional Challenges in Clinical Secretomics

The investigation of posttranslational modifications (PTMs) and their association with various diseases, including cancer, poses a further major challenge for secretome proteomics. The analysis of PTMs generally requires enrichment for modified proteins or peptides deriving from them to allow their detection in the face of commonly low abundance. Besides, proteomics research on PTMs has increased exponentially since the “open-search” approach was reported in 2015 (62). Implications for MS-based proteomics and recommended strategies have been recently discussed (42). Reverse phase protein arrays (RPPAs) (63, 64, 65) certainly represent the vanguard of PTM probing among affinity proteomic technologies in clinical samples (see further details below). Their detection of changes in protein modification is, however, strongly dependent on the availability of highly sensitive and specific antibodies.

For clinical study design, the choice of the ideal composition of a study cohort constitutes an additional challenge. Various confounders may severely affect secretome composition in addition to the general high intraindividual variability. Thus, inclusion and exclusion criteria, such as age, sex, disease state, medication, or presence of other diseases must be carefully considered.

A further challenge arising with the use of clinical samples is the possible introduction of preanalytical variation. Different protocols for sample collection (66), delay in processing (67), as well as storage may significantly impact measurable protein levels and must be considered prior to patient recruitment. Uniform standard operating procedures are paramount in order to ensure high and steady sample quality.

## Affinity Platforms for Secretome Studies

Many affinity-based proteomic approaches provide excellent sensitivity and specificity for low-abundance protein targets in body fluids. This is crucial also in their application to cancer, as relevant proteins (*e.g.* growth factors, cytokines, chemokines, and hormones), especially in early stages of the disease and in individuals with a low-tumor burden (52), typically fall into this low-abundance category. Semitargeted approaches may help to identify new diagnostic biomarkers and potential drug targets in this concentration range. Affinity technologies bypass the problem of potential biomarkers being masked by highly abundant coanalytes, particularly for detection in the context of blood. They also offer a high degree of multiplexing, allowing a large number of features to be analyzed simultaneously while using only small amounts of frequently limited sample material. Moreover, the assays have a wide dynamic range, capable of measuring protein levels of high- and low-abundant proteins in parallel, eliminating the need for extensive sample preprocessing, prefractionation, and depletion of high-abundant proteins prior to analysis. Furthermore, by using protein chips or multiwell plate formats, affinity proteomic technologies are operable in high-throughput, automated workflows, rendering them interesting for large clinical studies (Fig. 2). The latter aspect may be especially beneficial, for example, in validation studies, aiming to verify preliminary results from preclinical experiments using CM from cell or tissue cultures. In this context, standardized affinity-based protein assays further provide high flexibility regarding sample choice: many different sample types may be processed with only minor adaptions to the protocols. More precisely, the assays used in the flagship platforms of the two industry leaders SomaLogic and Olink use a series of dilution bins to address dynamic range. Olink Explore 3072, for example, uses five dilution groups for the analysis of serum and plasma samples. The dilution protocol may, however, be adjusted to allow better detection in sample types with inherently low protein concentrations, such as CM. Because of the broad range of cell culture conditions used, however, optimization for CM analysis necessarily represents an approximation.

The central downside to affinity-based technologies is their implicit restriction to a predefined panel of target proteins. Proteome coverage thus depends on the availability of affinity-binders such as antibodies or aptamers specific to the target proteins comprised. Despite such limitations, the assays are very powerful in settings where the proteins of interest are known from previous experiments and validation is aimed at in a larger cohort using midplex approaches. Their suitability further extends into high-plex screening in to ranges of protein concentration levels not easily reached using MS-based methods. Untargeted MS measurements on blood-derived samples (serum or plasma), commonly identify a few hundred to slightly above one thousand proteins (68, 69). In contrast, modern affinity proteomic technologies offer the analysis of thousands of features. For instance, SomaLogic, the current market leader with respect to panel size, provides 10,776 multiplexed protein assays, covering 9655 unique proteins (70).

**Fig. 3:**
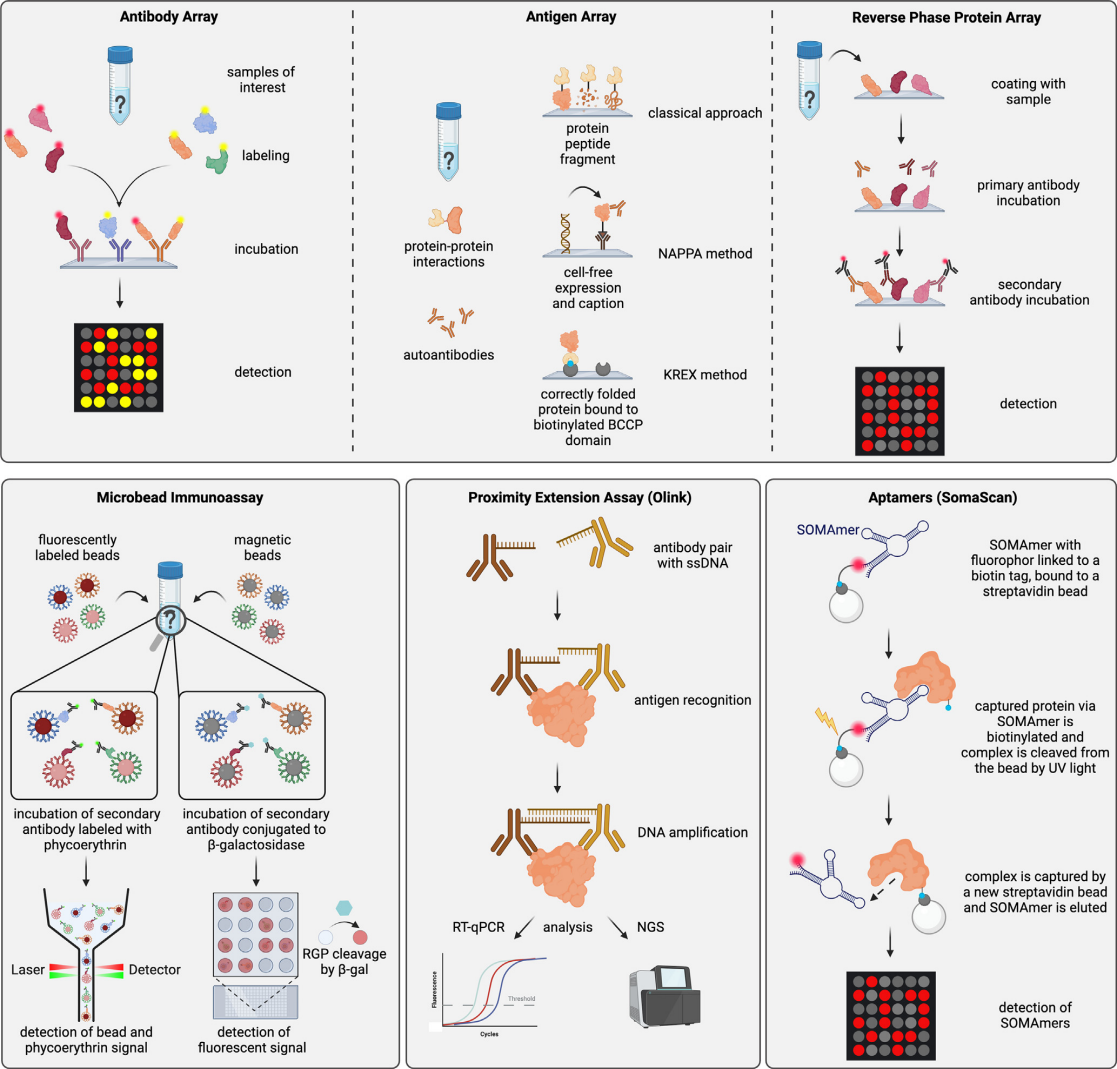
**Overview of the main affinity proteomics methods.** Key features involving the most common protein microarrays (antibody, antigen, and reverse phase protein arrays) are shown together with microbead immunoassay approaches as well as Olink (protein extension assay) and SomaScan (aptamer) technologies. Created with BioRender.

In any case, the methodology of choice ultimately depends on the research question addressed. MS- and affinity-based proteomics should thus not be seen as competing technologies but highly complementary methods (Table 1). MS excels at the analysis of cell and tissue samples, virtually independent of the model organism, trivially extends to the characterization of PTMs, and also provides highly accurate measurements of highly abundant proteins in complex matrices such as serum or plasma. In contrast, affinity platforms provide higher throughput and precise detection of low-abundant proteins in particular in matrices with massive dynamic range of protein concentration, which is highly advantageous in clinical proteomics. The following sections give an overview of the most commonly used and state-of-the-art affinity proteomic technologies (Fig. 3) and their application to tumor secretome analysis as covered to date by the scientific record.

**Table 1:** Main characteristics of featured proteomic platforms

Platform	Proteome coverage in blood		Limit of detection	Dynamic range	Sample -throughput/assay	Organisms	Key advantages	References
ELISA		singleplex	pg/mL	∼2 logs	∼40	all	minimal equipment, absolute quantification, high flexibility	(186)
								(187)
Luminex xMAP		∼500	pg/mL	4–5 logs	∼384	all	customizable, scalable	(111)
								(188)
SIMOA		singleplex, 6-plex panels	fg/mL	>6 logs	∼96	human	very high sensitivity, semi-automated	(189)
Protein		∼500	pg/ml – fg/mL	5–6 logs	<1056	all	flexibility (customization), cost-effective, PTM detection	(190)
Microarrays (RPPA)								(191)
Antibody		∼4000	pg/mL	5 logs	1–64	all	scalable, customizable, cost-effective, detect modifications	(192)
Arrays								(193)
Olink Explore		∼5300	pg/ml – fg/mL	10 logs	172	human, mouse	low sample consumption, high throughput, high sensitivity, semi-automated	(194)
								(195)
								(177)
SomaScan		∼11,000	pg/ml – fg/mL	10 logs	85	human	low sample consumption, high throughput, high sensitivity, semi-automated	(150)
								(70)
MS		∼400–6000 (w/fractionation and proteograph)	pg/mL	4–5 logs	Max. 18 (TMT)	all	untargeted, detect modifications, higher confidence in protein identification	(50)
								(184)
								(183)
								(68)

### Protein Microarrays

Protein microarray technology was adapted from DNA microarrays to achieve multiplex protein detection with high sensitivity. In high-density protein arrays, bait proteins are spotted or printed on a solid surface and immobilized *via* various binding chemistries coating the array slides. A plethora of forms exist, differing in the choice of immobilized bait. The most common types are antibody, antigen/functional, and RPPA. The choice of format is highly dependent on the individual research endeavor (Table 2). In general, forward phase arrays provide a higher number of features, while reverse phase arrays provide the analysis of a higher number of samples. In both versions, antibody capture of target proteins results in fluorescence signal, which may be recorded semiquantitatively. The signals are detected *via* fluorescence laser scanning devices or by charge-coupled device camera systems. Protein microarrays can detect even low-abundant proteins with high sensitivity with no need for depletion of highly abundant proteins.

**Table 2:** Application of protein microarray technology for the study of selected cancer entities

Cancer type	Sample type (number of specimens)	Purpose of the study	Proteome coverage	Technical information	References
Antibody arrays					

OC	Serum samples (OC patients, n = 34; healthy controls, n = 53)	Biomarker identification	174	RayBio Human Cytokine Array G-Series: G6, G7, G8 (RayBiotech)	(72)
	OC cell lines (SKOV3, CaoV3), patient-derived primary cells	Cytokine profile evaluation on tumor immune deficiency	79	RayBio Human Cytokine Antibody Array V (RayBiotech)	(196)
	HeyA8 cell line	Identification of CXCL8 signaling pathway regulation through ASS1	38	RayBio Human Chemokine Array C1 (RayBiotech)	(197)
PDAC	Target protein serial dilutions	Method development: Optimization of antibody arrays for biomarker detection	4	*In-house* customized, nitrocellulose-coated glass slides, piezoelectric contact printer (Auson 2470 Arrayer)	(73)
	Serum derived from PDAC patients (n = 39/n = 14)	Identification of serum cytokines as biomarkers/Prognosis significance of serum cytokines	507	Human Antibody L-Series 507 Array (RayBiotech)	(198, 199)
	Conditioned media of patient-derived cells (n = 3)	Impact of Galectin-1-induced upregulation of SDF-1	N/A	RayBio Human Chemokine Antibody Array Kit (RayBiotech)	(200)
	Conditioned media of PANC-1 cells	Analysis of secreted factors involved in cancer cell to pancreatic stellate cell interactions regarding ECM remodeling and cell invasion.	88	Human Cytokine Antibody Array (C5) + In-house array specific for EMT-related proteins	(201)
	Supernatant of dendritic cells and cytokine-induced killer cells	Validation of immune checkpoint CD40/CD40L activation improves DC action toward bile duct, pancreas and colorectal carcinoma	N/A	Cytokine Antibody Arrays (R&D Systems)	(202)
AML	K562, HS-5 cell lines	Impact of anticancer drug-treated stromal cells on K562 leukemia cells	79	RayBio Human Cytokine Antibody Array V (RayBiotech)	(203)

Antigen arrays/functional arrays					

OC	Plasma derived from healthy postmenopausal female donors (n = 9) and OC cancer patients (n = 9)	Evaluation of an autoantibody screening in plasma samples	90	Autoantibody Profiling System - 90 (APS-90; ITSI Biosciences)	(86)
	Serum derived from OC patients (n = 34) and matched controls (n = 30) in first stage	Evaluation of autoantibody detection with NAPPA arrays	5177	*In-house* array	(85)
	Serum derived from OC patients (n = 30) and matched controls (n = 30)	Identification of tumor antigen-associated autoantibodies for OC diagnosis	10,247	*In-house* array	(87)
	Supernatants of different cancer cell lines	Lectin microarray establishment for disease-specific biomarker analysis	45	Lectin microarray analysis by GlycoStation (Moritex)	(204)
PDAC	Serum derived from patients with PDAS, chronic pancreatitis, autoimmune pancreatitis (AIP-1 and AIP-2), gastro-intestinal diseases and controls	Development and evaluation of an autoantibody-screening for patients-derived serum	6.4	*In-house* array. Multiple spotting technique was used to synthesize proteins directly on the microarray slides (epoxysilane coated) according to Hufnagel et at. 2018 (205)	(88)
			345		
			36		
	Serum derived from intraductal papillary mucinous neoplasm, PDAC or control patients	Exploration of autoreactive antibodies in serum for identification of early-stage malignancy signatures	249	Produced and incubated according to Hufnagel et al 2018, (205). Epoxysilane-coated slides and noncontact Nanoplotter-2 (GeSIM) were used	(89)
	Serum derived from patients with PDAC, benign pancreatic diseases or matched controls	Identification of tumor-associated autoantibodies for PDAC diagnosis	21.216	HuPro Human Proteome Microarray v3.1 provided by CDI Laboratories and purchased from BC Biotechnology Co, LTD	(90)

RPPA					

PDAC	Serum or plasma derived from PDAC patients (n = 71), chronic pancreatitis patients (n = 30) or healthy controls (n = 48)	RPPA validation for biomarker screening in serum and plasma	1	tyramide-based signal amplification and colorimetric substrate detection by diaminobenzidine using Dako CSA kit (Streptavidin-Biotin Complex, Amplification Reagent, Streptavidin-HRP)	(91)
	Plasma derived from PDAC patients (n = 164) and healthy donors (n = 106)	Biomarker candidate identification in plasma samples	130	ProteoChip glass slides (Proteogen), antibody detection by peroxidase activity using Tyramide Signal Amplification (TSA) Cyanine 5 System (Perkin Elmer).	(101)
AML	Peripheral blood, leukapheresis, or bone marrow from newly diagnosed AML patients	Technique validation	>100	Nitrocellulose-coated glass slides, detection with DAKO signal amplification system, colorimetric detection of antibody-binding intensity 3,3′-diaminobenzidine tetrachloride cleavage by tyramide-bound horseradish peroxidase	(102)

#### Antibody Arrays

Antibody arrays are designed to detect proteins and quantify their relative levels in complex solutions. To this end, validated antibodies are spotted onto coated microarray slides and are surface-captured based on a range of different available binding chemistries. Samples containing the proteins of interest are labeled with a fluorescence dye prior to incubation on the arrays. Here, sample volumes of 10 – 100 μl for serum or plasma and 5–50 μg of EV protein in 100 μl are common. A common study design for antibody microarray studies is the usage of dual color arrays (Fig. 3). Here, samples from control and study groups are labeled with different dyes and incubated on the same array to reduce intraarray variability and allow for reciprocal internal control. Another approach is to incubate a standard reference sample along with the study samples, and use the former for data normalization, in particular across batches. Signal intensities are proportional to the protein level and allow a semiquantitative analysis. Given the availability of corresponding PTM-specific antibodies, this platform also allows for the detection of posttranslationally modified proteins. One limitation of antibody arrays is the common restriction to a single antibody per target. However, sandwich-type microarrays, such as reverse capture antibody arrays (71), which in essence represent a miniaturized ELISA, may be found. While such approaches imply increased sensitivity and render labeling of samples obsolete, they are also associated with significant cost. Commercially available arrays with several thousand unique features are less economical as compared to other high-plex methods such as proximity extension assay (PEA) or aptamer panels described below. High-density antibody arrays are suitable for biomarker discovery and exploratory studies and have been used in different secretome profiling studies of blood-derived samples as well as cell culture supernatants. In the case of OC, markers such as CA125, macrophage stimulating protein alpha (MSP-α), platelet-derived growth factor receptor alpha (PDGF-Rα), osteoprotegerin (OPG), and metalloproteinase inhibitor 4 (TIMP-4) were identified by antibody arrays analyzing serum samples (72), while hippocalcin-like protein 1 (HPCAL1), phosphatidylethanolamine-binding protein 1 (PEBP1), galectin-7 (LGALS7), and serpin E2 (SERPINE2) were defined for PDAC patients (73) (Table 2). An additional example for a secretome study using this technology is the analysis of cell culture-derived supernatants in the context of OC, PDAC, or acute myeloid leukemia (AML) (Table 2). Interestingly, initial multiplexed studies aiming to molecularly phenotype EVs were performed using antibody arrays using antibodies against tetraspanins CD9, CD63, and CD81 (74) to capture small EVs. This technology was further developed (75) and eventually commercialized in the product EV Array (supplied by Scienion), which has been used, for example, to study non—small cell lung cancer (NSCLC) (32, 76) and ovarian cancer (77). Further comparable EV studies include head and neck (78), bladder (79, 80) and breast (81) cancer entities.

#### Antigen Arrays

Antigen or functional microarrays are used to investigate protein-protein interactions, including screens for autoantibody profiles (Fig. 3). In this type of microarray, full-length proteins, protein fragments, or peptides are spotted and immobilized on functionalized microarray slides using a broad range of surface coatings. Besides the classical approach, multiple methods have been developed to approximate physiological antigen presentation on the slide. Nucleic acid programmable protein array, reviewed in (82), is one such approach. Nucleic acid programmable protein array uses cell-free expression systems to produce the respective proteins *in situ*, on the array slide from prespotted complementary DNA molecules. This method circumvents the need for protein expression and purification from *in vitro* or *in vivo* systems and provides a consistent and reproducible presentation of the antigens on the array slides. In contrast, standard immobilization methods may lead to protein denaturation allowing only linear epitopes to be accessible for detection. This particular challenge is also tackled by the KREX technology offered by Sengenics (Sengenics, KREX, Protein Folding Technology. Available at: https://sengenics.com/technology/krex-protein-folding-technology/). Here, full-length proteins are fused to the biotin carboxy carrier protein domain of acetyl-CoA carboxylase using an insect cell expression system. Only if the produced protein attains its native conformation, the biotin carboxy carrier protein domain is correctly folded as well and subsequently available for biotinylation. Accordingly, downstream immobilization of fusion proteins on streptavidin-coated slides implies a native state. Of commercially available platforms, the HuProt Proteome Microarray currently provides the broadest coverage of human proteins and contains more than 21,000 full-length proteins, isoform variants, and protein fragments on one slide (CDI Labs, cdi.bio).

As an abundant and important component of the blood secretome, B cell-secreted antibodies are frequently investigated as disease biomarkers. The focus of such research is the detection of special autoantibody profiles that change in relation to pathogenesis. In this context, it is noteworthy that pathology may result in a broad spectrum of protein alterations like PTMs, truncations, mutations, aberrant expression profiles, or misfolding, all potentially eliciting an immune response (83). Such altered profiles have also been observed in malignant disease, and have, in turn, been termed tumor-associated antigens (TAAs), with corresponding antibodies already detectable at early disease stages and therefore promising high utility in cancer diagnosis (84). Autoantibody profiling using antigen microarrays thus provides an indirect analysis method to detect TAAs in the tumor secretome. As such, it has been successfully applied in studies of various cancer types such as OC (85, 86, 87) or PDAC (88, 89, 90). Remarkably, both custom, cell-free expression (88, 89) or HuProt antigen microarrays (90) have been demonstrated to be powerful platforms for plasma analyses aiming to better stratify PDAC patients not only against benignant diseases but also against other inflammatory conditions (*e.g*., chronic pancreatitis). A major advantage of antigen arrays for TAA-associated autoantibody detection lies in the high specificity and detectability in serum long before disease diagnosis. In OC, for example, a panel of 11 TAA-associated autoantibodies was able to distinguish patients from healthy controls with a sensitivity of 45% at 98% specificity (87), while a three marker panel showed a sensitivity of 23.3% at 98.3% specificity (85). These potential biomarkers hold large potential in early cancer detection and therefore improved patient outcome.

#### Reverse Phase Protein Arrays

RPPAs were designed to quantify protein expression in cell lysates but may be applied to practically any biological liquid sample. In comparison to the forward phase array systems, RPPAs are generated by directly immobilizing the protein sample on the surface of a microarray slide. Slides are further incubated with a specific primary antibody, followed by detection *via* a dye-labeled secondary antibody, analog to conventional WB (Fig. 3). Samples are usually printed onto the array surface in a dilution series to enable protein quantitation across a linear range that can be prepared from minute sample volumes as small as 1 μl (91). A frequently used approach to calculating single expression values from the dilution curve, firstly introduced by Paweletz *et al.* (92), is the application of a dose interpolation algorithm as suggested by Nishizuka et al (93). The method has now been used for more than 20 years and has been constantly improved since its inception in the early 2000 s. Currently, RPPA platforms are used to quantify protein levels in a large number of samples while only using small volumes of analyte. One major advantage of the method is its flexibility and cost efficiency. Many different sample types can be analyzed using the platform, ranging from cell and tissue lysates to formalin-fixed paraffin-embedded samples; however, preanalytical variation majorly impacts data quality. One of the challenges in using this method is the availability of highly specific, validated antibodies. In contrast to WB, unspecific binding to proteins other than the target protein cannot be identified in sample micro-spots. It is therefore required for antibodies used to show a single dominant band in WB, ideally with further validation through a second method such as ELISA. Likewise, it is important to mention that the availability of this method to the research community is unfortunately limited as currently no RPPA is commercially available. RPPAs require customization for every experiment with specific demands for various sample types and few laboratories are positioned to consistently handle large amounts of samples, limiting the access to researchers. Nonetheless, RPPA has been applied for the proteomic profiling of cell lysates (94, 95) and tumor specimens (96, 97, 98), as well as organoids (99) and serum proteomics (100). Particularly in cancer, RPPAs have been used for general protein expression as well as signaling pathway analysis in tumor cells and biopsies, and to a less extent to secretome samples (Table 2). The method has been widely applied to study molecular effects of novel treatments or combinations of existing therapy regiments, as it may not only provide information on protein expression levels but also on pathway activation by consideration of differential phosphorylation states of signaling proteins. A particularly interesting application of RPPA is the investigation of blood protein levels as sero-diagnostic markers. In PDAC, RPPA has, for example, been applied to validate CA19-9 as monitoring blood biomarker, thereby proving its value in the high-throughput screening of biomarker candidates (91). As CA19-9 alone is insufficient for the early detection of pancreatic cancer, other researchers investigated new biomarkers that could improve the diagnostic potential. Yoneyama et al. examined 130 proteins as candidates and found that blood levels of insulin-like growth factor-binding proteins 2 and 3 (IGFBP2 and IGFBP3) in combination with CA19-9 significantly increased test performance for early stage invasive ductal adenocarcinoma of pancreas compared to CA19-9 alone (101). The method has also proven to be a sensitive and reproducible tool to analyze protein expression levels in peripheral blood samples from AML patients in parallel with bone marrow specimen (102). RPPAs have also demonstrated their value in clinical studies of other cancer entities. An optimized RPPA assay platform was applied to quantify the serum protein expression of 10 putative biomarkers of hepatocellular carcinoma (HCC), six of which showed high discriminatory potential between HCC and healthy controls (100). Evaluation of serum protein levels is also of interest for monitoring wound healing and a possible relapse after cancer surgery (103). Diagnostic serum markers are also in high demand for the early detection of lung cancer, and two strong biomarker candidates, cytoskeleton-associated protein 4 (CKAP4) and calnexin (CANX), have been identified using RPPAs (104, 105). EV research has also benefited from application of RPPA. One study, for example, comparatively analyzed the protein composition of small plasma EVs from healthy donors and breast cancer patients at different disease stages before and after surgery, identifying FAK and fibronectin proteins as markers of high diagnostic and therapy response accuracy (106). Other examples include the study of sera from prostate cancer patients where differentially expressed end points (*e.g.,* programmed death-ligand 1 (PD-L1), CD274), survivin, and transforming growth factor beta were identified as deriving from T-EVs (107).

Altogether, these results highlight protein microarrays as powerful tools in the elucidation of modes of action of existing treatments and/or to reveal alternative potential drug targets involved in similar pathways, opening new therapy options.

### Microbead Immunoassays

In place of antibody immobilization on a planar surface, bead-based assays use microbeads to carry capture antibodies. The beads are incubated in suspension with the sample and captured proteins detected by a labeled secondary antibody. One of the most widely used assays of this class is the Luminex xMAP (x–Multi-Analyte Profiling) technology, which is frequently applied to cytokine detection in serum and plasma samples. Luminex beads are marked with individual ratios of red and infrared fluorophores, enabling multiplexing. Beads labeled with a specific fluorophore ratio are coated with a target-specific antibody and, subsequently, multiplexed bead mixtures generated. Following incubation with the sample, the proteins of interest are detected by biotinylated secondary antibodies and a streptavidin-phycoerythrin reporter. The assay read-out requires detection using multiple lasers, with one wave length identifying the unique bead signal (reflecting the specific antibody/target complex), and another one detecting the phycoerythrin signal proportional to the respective protein concentration (Fig. 3). With the most advanced Luminex platform, FLEXMAP 3D, up to 500 proteins may be analyzed simultaneously with >4.5 logs dynamic range (Table 1). Luminex assays have been applied to identify cytokine, growth factor, and chemokine profiles in many different malignancies and other diseases (108, 109, 110, 111, 112). Luminex assays are approved by the U.S. Food and Drug Administration (FDA), rendering them also applicable for clinical diagnostics. Quanterix offers the single-molecule array (SiMoA) technology as a platform for biomarker discovery in different sample types (113). In a recent comparative study of several popular immunoassay formats, this platform showed superior overall performance and shall therefore be further highlighted here (114).

#### The SiMoA Platform

This detection method uses single-domain antibodies coupled to paramagnetic beads, which are incubated with the liquid sample. Target proteins bound on the bead surface are detected by a secondary antibody, conjugated to β-galactosidase capable of cleaving the substrate resorufin β-D–galactopyranoside, generating a fluorescent signal. Antibody beads are added in excess so that, according to the Poisson distribution, they will capture a single protein target molecule or none. The samples including the bead anchored immunocomplexes are subsequently loaded onto a so-called SiMoA disc, providing an array with 200,000 fl-volume microwells, which each may hold exactly one bead. Fluorescent images capture signal from the array, at each location generated by a single immunocomplex. The protein concentration in solution correlates with the percentage of beads generating a signal (115, 116). This method is highly effective in detecting proteins at very low concentration down to fg/ml (Table 1). With the SiMoA HD-I and HD-X Analyzer, Quanterix provides a fully automated analysis platform ensuring high reproducibility and accuracy (117). Targets may be selected from an assay menu currently covering in excess of 80 target proteins. Multiplexing is, however, limited to ≤10-plex measurements, depending on the assay kit. This renders SiMoA assays unfeasible for early large-scale screening studies. The technology holds, however, great potential for validation studies of small marker panels further downstream the biomarker pipeline (Fig. 2). The available *Homebrew* (https://www.quanterix.com/products-and-services/homebrew/) option to develop 4-plex assays with customizable target panels may, in particular, be suitable for late-stage validation studies.

#### Application of SiMoA Platform in Tumor Research

Due to its high sensitivity, which even outperforms ELISA as the gold standard technology platform, SiMoA technology has been widely used in cancer research (118, 119) for the detection of secreted proteins at ultra-low concentration in serum or plasma samples (Table 1). In a mouse prostate cancer xenograft model, Schubert et al. demonstrated elevated serum PSA levels to be detectable at significantly earlier stages as compared to standard and ultrasensitive ELISAs (118), and quantified PSA levels even from single prostate cancer cells (120). The assays have further been used to evaluate squamous cell carcinoma antigen as a predictive serum biomarker for status and treatment response for cervical cancer (121), interleukin (IL)-12p70 as a predictive biomarker in cancer immunotherapy (122), and to investigate leucine-rich repeats and immunoglobulin-like domain protein 1 (LRIG1) as a prognostic marker in OC (123), reaching very low detection limits in the picogram range. While the assay focuses on the analysis of serum and plasma samples, the technology has also been used for other matrices, *e.g.*, CSF (124), sweat (125), tears (126), CM, or cell lysates (119).

Interestingly, SiMoA assays have not only been used to detect single protein molecules but also been applied to directly detect and quantify EVs in body fluids *via* EV-specific membrane-bound proteins and tumor surface markers. Using this approach, CD9-and CD63-positive EVs in plasma samples were, for example, quantified in breast cancer patients using respective detection antibodies, and showing that higher EV plasma levels are significantly associated with tumor presence (127). Additionally, EVs from plasma and cancer cell culture supernatants were studied in the context of colorectal cancer (128). Here, a combination of EpCAM/CD63 and CD9/CD63 capture antibodies was used to detect tumoral (T-EVs) and total EV levels, respectively. Other groups have developed different SiMoA EV detection assays using PD-L1/CD63 detection antibodies to specifically detect T-EVs in comparison to canonical EV markers, as PD-L1 was shown to be more abundant in T-EVs (129). Further combinations using EpCAM/PD-L1 detection antibodies were tested to analyze EVs in lung cancer (130, 131). It is noteworthy that PD-L1 expression in tumor cells can inhibit T cell proliferation, thus promoting immune evasion of cancer cells. The use of the SiMoA technology to prove PD-L1 presence on EVs may also be a viable approach to predict tumor PD-L1 expression levels, and potentially stratify responders and nonresponders of immune checkpoint inhibitor therapy in a noninvasive manner (132).

SiMoA assays do not provide a sufficient degree of multiplexing for novel biomarker screening among secreted proteins but have the potential to verify smaller panels with high sensitivity already in early-stage cancer with low tumor burden. A number of SiMoA assays have already been approved by the FDA as diagnostic and prognostic aids, which shows their potential as tools in clinical settings. Current efforts aim at enhancing the limits of detection in SiMoA assays even further with the development of droplet digital ELISAs. Droplet digital ELISAs technology is also based on single-molecule protein detection, but able to measure protein concentrations in the attomolar range (133, 134), which may tremendously improve early cancer detection.

### Proximity Extension Assay (Olink)

**Fig. 4:**
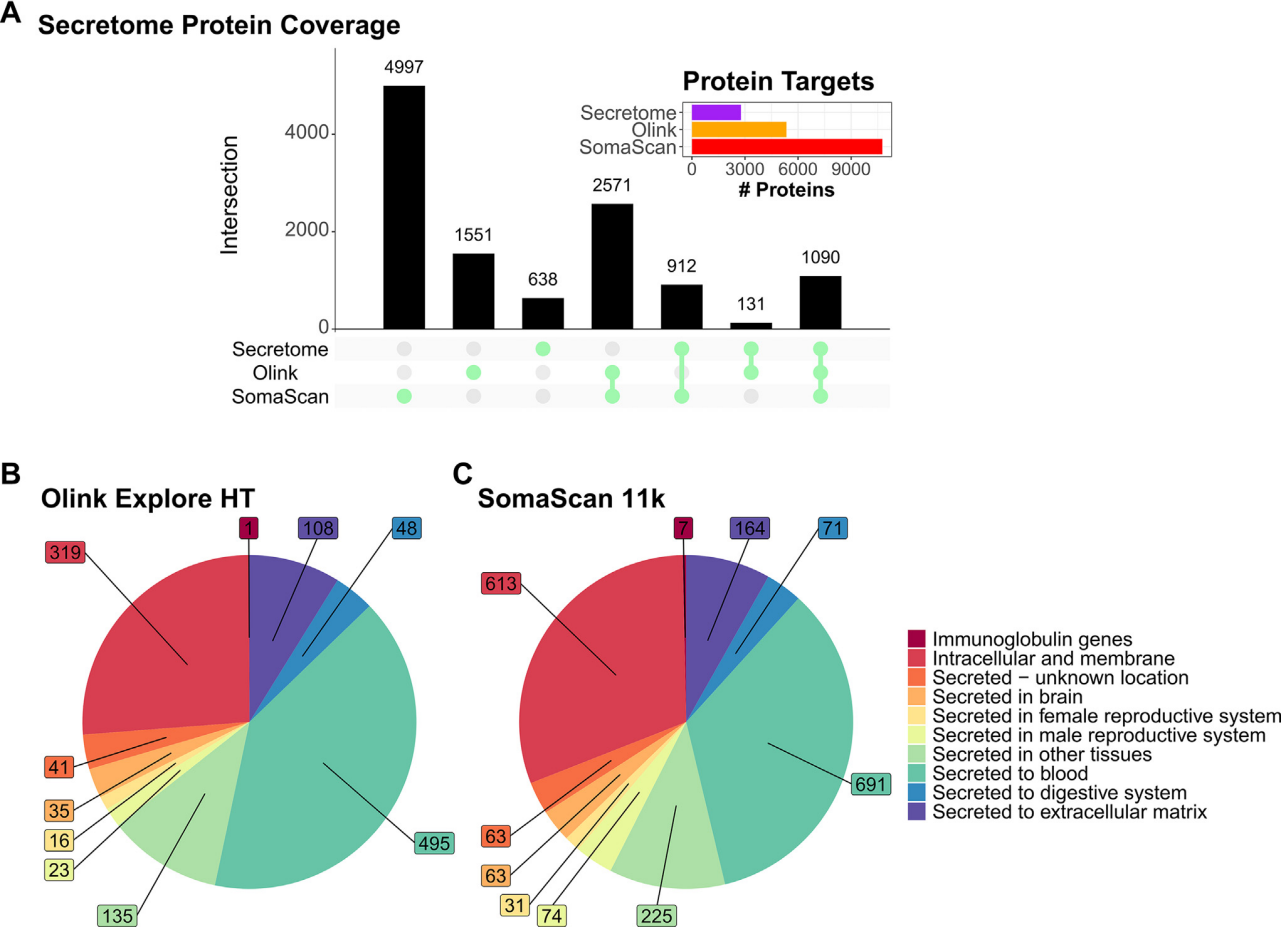
**Coverage of predicted secretome proteins using Olink HT and SomaScan platforms.***A*, UpSet plot of secretome coverage comparing the two largest affinity proteomic platforms Olink Explore HT and SomaScan. Overall protein count is depicted in the inlay plot, with predicted secretory proteins (*purple bar*), Olink Explore (*orange bar*), and SomaScan (*red bar*). Counts of human secretome proteins are based on HPA definition (https://www.proteinatlas.org/humanproteome/blood+protein) (8). *B* and *C*, pie charts showing the compartment of predicted secretory proteins covered by Olink Explore HT (*B*) and SomaScan 11k (*C*). HPA, Human Protein Atlas.

The PEA represents an immunoassay approach in which two antibodies bind simultaneously to two different epitopes on the same target protein, similar to an ELISA (Fig. 3). Each of these antibodies is covalently modified on their Fc domain with a complementary ssDNA probe, which hybridizes only when the correct pair of antibodies binds in each other’s proximity. The so-formed short dsDNA sequences are preamplified by PCR, followed by a subsequent readout *via* quantitative real-time PCR or next-generation sequencing. The resulting DNA sequence is unique to every protein target and its quantity is proportional to the protein concentration in the sample. Thus, protein levels are reported as relative protein abundance. The design of PEA, with minimal cross-reactivity and high specificity, allows for a high degree of multiplexing across a broad dynamic range (10 logs) and is suitable for high-throughput analyses. Olink has commercialized this technology and offers it as mid- (Olink Target) and high-plex (Olink Explore) platforms. The latest version, Olink Explore HT, offers the parallel analysis of 5343 unique targets from 2 μl sample (Fig. 4 and Table 1). The Explore workflow generates normalized protein expression values by which the proteins can be relatively quantified. In the case of the Olink Target, absolute quantification may also be performed.

Notably, PEA is highly scalable and has the potential to in the future analyze even more protein targets simultaneously. While PTM-specific antibodies are generally available, the PEA technology currently does not enable the analysis of PTMs and the implementation of this feature remains a future prospect. Additionally, the implementation of assays using antibodies to recurrent mutations in tumor entities could highly expand the utility of this platform in cancer diagnostics.

#### Application of Olink Platforms in Tumor Research

Olink’s Explore and Target panels were designed for the analysis of human serum and plasma samples. However, a wide range of different matrices can be analyzed using this workflow, including sample types with low protein yield by adjustment of the utilized dilution groups. Successful applications include various body fluids, such as tears (135), saliva (136), aqueous humor (137), urine (138), CSF (139) and more. Beyond this, preclinical sample types as cell culture media and cell lysates may also be processed, highlighting the assay’s flexibility to investigate different secretome profiles. For example, the recent application of PEA-based approaches for the analysis of the secretome of primary T cells has helped to understand the role of T cell-derived tumor necrosis factor alpha (TNFα), IL-2, and IL-21 in cytolytic activation of natural killer cells in the context of OC (140), validating their clinical relevance previously reported (56, 141). The technology has also demonstrated good performance in early stage cancer detection from plasma samples in a recent proof-of-concept study, able to even classify different types of cancer (142). In contrast, a recent, large prospective study failed to identify proteins with the potential to improve prediction of short-term breast cancer risk among the targets tested (163 assays), as observed effect sizes were too small to produce significant results after correction for multiple testing (143). Nominally, significant associations of the protein levels still provided valuable insight into the molecular mechanisms underlying cancer.

#### Application of Olink Platforms in EV Research

PEA has also proven valuable for the analysis of the EV compartment of the secretome. Olink Target panels were applied for the analysis of plasma and lysed EVs isolated from the blood of patients with myocardial infarction (144). In another study, EVs were isolated from CSF using four different extraction methods and analyzed using two different PEA panels comprising 92 targets each (139). Isolation methods in this study had a major impact on the detectable protein profiles, which should be considered when comparing EV proteomic studies.

The study of EV protein composition is of increasing interest for the identification of early cancer biomarkers and the assessment of treatment response. In a recent study, Viktorsson et al. analyzed the protein expression in plasma-derived EVs of metastatic urothelial carcinoma patients using the Olink Target Oncology II panel to identify patterns that are indicative of treatment success (145). With the assistance of a machine learning algorithm, they discovered protein patterns associated with progression-free survival and superior treatment response. Moreover, PEA has further been applied to identify brain tumor-associated protein signatures in plasma-EVs as biomarkers for diagnosis and disease progression (146, 147).

Notably, PEA is highly scalable and has the potential to analyze even more protein targets simultaneously. While PTM-specific antibodies are generally available, the PEA technology currently does not enable the analysis of PTMs and the implementation of this feature remains a future prospect. Additionally, the implementation of assays using antibodies to recurrent mutations in tumor entities could highly expand the use of this platform in cancer diagnostics.

### Aptamers (SomaScan)

Approximately 30 years ago, the use of aptamers as specific protein ligands was first described (148, 149), and since then the technology has been constantly developed and optimized (150). Aptamers are short, synthetic oligo ssDNA or RNA nucleotides with the ability to, based on their three-dimensional structure, specifically bind proteins or other molecules (Fig. 3). Aptamers are selected through a process called systematic evolution of ligands by exponential enrichment (SELEX), in which a large library of random oligonucleotides is iteratively selected for binding to a target molecule (151). After several rounds of selection and enrichment for oligonucleotides with high affinity and specificity for the target molecule, the oligonucleotides are sequenced to identify their specific sequences. Currently, the most used aptamer-based assay platform is the SomaScan technology commercialized by SomaLogic Inc. It uses slow off-rate modified aptamers (SOMAmers) as enhanced aptamers, including nucleotides chemically modified to mimic amino acid side chains to increase affinity to protein targets (152). SOMAmers are conjugated to a fluorophore and a biotin tag *via* a photocleavable linker and bound to streptavidin beads prior to incubation with the sample. The captured proteins are biotinylated themselves prior to cleavage of protein-aptamer complexes from the beads *via* UV light exposure. The complexes are recaptured on fresh streptavidin beads and the SOMAmers eluted and analyzed on a microarray chip. The intensity of the signal from the respective fluorophores is proportional to protein concentration in the solution. With approximately 11,000 target proteins to be measured in 55 μl sample (SomaScan 11K Assay), SomaLogic provides the largest currently commercially available affinity proteomics–based assay (Fig. 4 and Table 1).

#### Application of Aptamer-Based Technology in Tumor Research

Similar to Olink PEA technology, SomaScan is validated for the analysis of serum and plasma samples but may also be applied to a plethora of different body fluids like urine (153), stool protein extracts (61), CSF (154), or ascites peritoneal fluid (56). Even proteomic analysis of cell lysates is feasible using the SomaScan technology, giving new insight into chemotherapy treatment dependent on alterations in the B cell proteome in lymphoma (155). The assays have been further used for proteomic studies, biomarker detection, and validation in different cancer types, including lung cancer (156, 157), HCC (158), OC (159), pancreatic cancer (5), and many more. Efforts to analyze tumor microenvironments using liquid samples as analytes have also been made, and a significant upregulation of CCL23 expression in bone marrow aspirate samples of AML was shown using this approach (160). Since not only protein abundance but also protein modifications may be altered in different malignancies, the identification of PTMs has the potential to add valuable information to corresponding proteomic analyses (161). With that goal in mind, aptamers are also being developed to discriminate different PTMs such as glycosylation (162) or phosphorylation (163). Commercial availability of aptamer panels including such reagents remains, however, elusive.

#### Application of Aptamer-Based Technology for EV Studies

SomaScan assays have further proven valuable in the discovery of EV-associated cancer biomarkers (164). This study identified over 300 EV proteins associated with prostate cancer and further discovered novel proteins showing enrichment in blood-derived EVs (164). The potential to use this technology for the discovery of noninvasive disease markers in EVs has further been confirmed by a study investigating the EV-associated proteomes in plasma and urine from prostate cancer patients (165). They showed that of 1000 probed proteins, about 400 were detectable in similar quantities in vesicles from both sample types.

## Implementing and Upgrading Secretomics

MS-based and affinity-based proteomics should not be seen as adversary but as complimentary tools. Combinations of both have been widely applied in different formats and the potential synergy between the approaches continues to increase. Classical workflows with MS-based discovery and subsequent validation using ELISA or WB have long since proven robust, but combinations of MS and the novel PEA or aptamer-based platforms are also proving viable (56, 166, 167).

In general, affinity proteomic platforms are targeted approaches and not hypothesis-free. This aspect is more prominent in low- and mid-plex formats, while platforms covering several thousand target proteins provide broader insight into many different biological processes. Also, a *de novo* identification of cancer neoantigens is not possible and detection of PTMs is limited due to a lack of available binder molecules. That aside, microarray platforms are, for example, not the best choice for exploratory screening studies, as they provide lower plex analyses at high cost and limited throughput. However, microarray protein technology becomes very advantageous when a list of candidate protein biomarkers has already been established or only specific biological processes are targeted, as they are highly flexible and may be tailored to answer very specific questions.

Also, commercially available bead-based arrays have a narrow menu of available target analytes and are thus of limited use to broad, exploratory analysis of the secretome in the context of various diseases, as well as for the discovery of novel biomarkers and drug targets. Their simplicity of use and overall high precision, however, render them an interesting option to be applied in clinical settings, especially when official approvals from agencies, such as the FDA, are required.

### Extending the Secretome Coverage by Affinity-Based Methods

The current generation of high-throughput, high-plex affinity proteomic platforms, using antibody- or aptamer-based protein detection, provide workflows with a high degree of automation and may be efficiently used for the exploratory study of thousands of proteins. Interestingly, they have also been shown to be of high utility in combination with genomics data in large-scale proteogenomics studies, resulting in, for example, the identification of novel protein quantitative trait loci (pQTLs) (168).

When comparing the two leading platforms regarding the coverage of the secretome, defined as an overlap with the HPA (Available at: https://www.proteinatlas.org/humanproteome/blood+protein), the extensive protein list of the SomaScan platform renders it the front runner (Fig. 4*A*). While 1090 protein targets are covered by both platforms, SomaScan out-competes Olink with respect to unique HPA targets by a margin of 912 *versus* 131 proteins. It should be noted that, approximately 1/5 of HPA-categorized secretome proteins remain not covered by either of the platforms (638 proteins). From a different perspective, when comparing the proteins annotated to different cellular compartments, SomaScan covers more targets in all defined subproteomes, while the relative distribution of target coverage is similar in both platforms (Fig. 4, *B* and *C*).

In a direct large-scale comparison, technology performance was assessed with particular regard to protein targets found in both and their association with cis pQTLs from existing genomics data (169). Here, the SomaScan platform had an overall higher precision with lower coefficient of variation (CV), while Olink PEA showed a higher target specificity. The correlation between overlapping assays on each platform was classified into three categories: very high correlation, nearly no correlation, and mediocre correlation. Low and medium correlations were suggested to be caused by the corresponding platform not measuring the same protein or by being differentially influenced by PTMs or epitope accessibility. Proteins on the Olink platform showed more pQTLs, notably also among shared targets with low correlation, indicating that the PEA technology is more likely to detect the intended protein. This may be related to differences in the protein detection between the two methods. The SomaScan technology is based on single epitope recognition mediated by one aptamer per target protein. In contrast, the Olink Explore technology is based on dual epitope recognition, involving two antibodies per target protein. Another study analyzing 1514 plasma samples from an Icelandic cohort on both the Olink Explore 3072 and the SomaScan v4 platform came to similar conclusions (170). Protein targets measured in both platforms were integrated with preexisting genomics data to identify pQTLs associated with altered protein levels. The two platforms were also indirectly compared based on SomaScan data from 35,892 samples of the Icelandic cohort and Olink Explore 3072 data from 54,265 UK Biobank participants. Both platforms shared an overlap of 1823 proteins. Consistent with the results from Katz *et al*. (169), evaluation of the precision from repeated measurement of the same samples (Olink: n = 1474, SomaScan: n = 227) in this study revealed overall higher CV for the Olink assays compared to SomaScan assays targeting the same protein (median CV 14.7% *versus* 9.5%). The median correlation between shared assays on both platforms was considerably low, reaffirming the results of other studies (169), but also differed vastly between individual assays, depending on sample dilution and expected detectability in plasma. Combination of the proteomics data with genomic data allowed the identification of a large number of pQTLs, however, there were again differences between Olink and SomaScan in their detection. Particularly the number of detected cis pQTLs was higher with the Olink Explore assay, but a large number of pQTLs could be observed in both platforms uniquely (170). The varying correlation between the platforms was also observed in other studies and median Spearman correlations of ρ = 0.33 to 0.73 have been reported for overlapping assays (56, 169, 171, 172, 173). This indicates that the two technologies are likely to target different proteoforms. Correlations of SomaScan and Olink assays in comparison to MS-generated data were slightly better than what is generally reported (ρ > 0.7), even though only a small number of samples was evaluated (174). Moderate correlation of Olink PEA was further observed in ELISA validation assays (ρ > 0.5) (171). For all affinity-based proteomic approaches, altered epitopes may affect binder affinity, rendering the unambiguous identification of proteins under all circumstances difficult. Also, cross-reactivity with other proteins may never be entirely ruled out, even though the binders undergo multiple iterations of selection and validation. Cross-reactivity is also a major challenge when it comes to the analysis of cell culture media containing bovine serum proteins, which needs to be accounted for, resulting in higher LODs. Nonetheless, both technologies are very powerful for the discovery of low-abundant proteins from liquid biopsies, including those addressed in cancer studies, revealing different facets of the proteome.

Availability of clinical samples is often limited. Therefore, considerations of sample consumption may be a factor of high importance. Depending on available resources, the trade-off between depth of protein coverage and sample management needs to be considered. As standardized assay conditions for highly specific sample matrices with an extensive range of formulations as CM cannot be provided, sample requirements are outlined for plasma in the following. As an example for cutting-edge affinity-enrichment workflows for MS-based proteomics, the Proteograph XT workflow, discussed below, requires an input of 240 μl plasma and can provide identification of up to ∼5000 protein groups when coupled with state-of-the-art DIA LC-MS (175, 176). Depending on experiment design and available setup, antibody microarray approaches usually require 10–100 μl of plasma, while RPPAs can be prepared with 1 μl, covering a highly setup-depending number of target features (72, 73, 78, 91). The SomaScan platform can identify up to 9655 proteins in a sample input volume of 55 μl (70), whereas 10 μl of sample are required for Olink Explore HT to analyze 5343 proteins (177). Proteomic analyses using neat plasma samples or other preparation kits may require less sample input at a reduced cost, while also yielding strongly reduced numbers of protein identifications (<1000) (167, 178).

Recent state-of-the-art MS with or without combination with affinity-enrichment, as provided through the Proteograph suite, has proven to be complementary to the affinity-based platforms (56, 166, 167). The combination of technologies enables exploration of different proteome facets through identification of proteins exclusively detectable with single platforms and can compensate for disadvantages of either approach (167, 176). Major advantage of affinity-based approaches is the high sensitivity for very low-abundant proteins not detectable by MS, while MS-based workflows provide unbiased results that are not affected by potential epitope effects (179). MS and affinity-based proteomics run in parallel can circumvent common caveats in each analysis method and can be used to complement each other to get more comprehensive proteomic data.

### Emerging Platforms

In addition to the well-established affinity-based technologies described above, two new promising approaches to study the secretome have recently become commercially available and deserve a brief introduction, as we see great potential for their future application in biomedical research.

#### NULISA (Alamar)

Alamar developed a new ultrasensitive immunoassay format based on proximity ligation assays (PLAs) (NULISA Alamar Biosciences. Available at: https://alamarbio.com/technology/nulisa-platform/) (180). PLA was a predecessor of the PEA technology used in the Olink platform and is similarly based on dual epitope recognition using antibody pairs conjugated to oligonucleotide probes. The major difference between the two approaches is that in PLA a DNA ligase instead of a DNA polymerase is used to yield the nucleotide amplicon quantified in proxy of the protein abundance. Originally, ligase dependence was replaced in favor of a polymerase reaction for PEA based on the observation that plasma samples interfere with the DNA ligase activity in standard PLA (181, 182). NULISA overcomes this problem by the introduction of two bead-washing steps prior to the ligation reaction, which concomitantly increases the signal-to-noise ratio. During NULISA assay development, signal fine-tuning is performed, using a “hot/cold antibody mixture”. Here, nonsignal antibody pairs are introduced to reduce reads from high-abundant proteins and increase dynamic range. Similar to PEA, the platform uses next-generation sequencing as a readout method for multiplexed assays, enabled by sample and target-specific barcoding of the DNA strand. It provides full automation and achieves sensitivity in the attomolar range, showing a 10-fold lower LOD as compared even to the highly sensitive SiMoA assays and might thus provide a higher sensitivity for low-abundant biomarkers also compared to Olink Explore (PEA). Currently, Alamar offers two panels, covering central nervous system disease biomarkers (>120 targets) and inflammatory proteins (>250 targets), which render them interesting to novel application in tumor research.

#### Proteograph (Seer)

MS-based methods are also playing catch-up with the affinity proteomic market leaders, reaching new depths in proteome coverage from human body fluids. One particularly ambitious approach will be briefly highlighted here. The fairly recent Proteograph XT workflow offered by Seer (175) (https://seer.bio/products/proteograph-product-suite/) combines an affinity enrichment of plasma/serum proteins with classical MS-based analysis methods (183). The platform offers more than 275 different nanoparticles (NPs) with unique physicochemical properties, of which a panel of five is used in the Proteograph XT suite. Here, proteins bind to the NPs based on their affinity to the functionalized surface, creating a corona of proteins and rendering low-abundant proteins relatively more abundant. Captured targets are directly digested on the NP, and, after purification and drying of the peptides, may be analyzed using standard MS instrumentation. Sample preparation is fully automated using the SP100 automation instrument from Hamilton. Recently, this enabled the identification of more than 6000 proteins in human plasma samples, covering a broad concentration range (184) and another recent study reported an up to 4.5-fold increase in amount of identified peptide precursors on the timsTOF HT (Bruker) with the Proteograph suite compared to neat plasma samples (185). The Proteograph XT suite was used in a recent pQTL study, reporting the identification of ∼3000 proteins in >320 plasma samples (176). Epitope-altering genetic variants can bias affinity-based assays for pQTL identification. Therefore, the authors compared the pQTLs from their MS-based analysis with previously discovered associations based on SomaScan and Olink Explore. The authors reported an overlap of two thirds, further affirming the specificity of the affinity binders used in the two platforms, while also identifying novel pQTLs that were not reported in either study. In a recent preprint, however, the depth of the proteome coverage achieved with the Seer platform was relativized: While a large number of 5753 proteins could be quantified across the complete study cohort of 1260 participants, only 1980 proteins were quantifiable in >80% of samples (179). The clear advantage of this platform relies on the untargeted protein detection and the analysis on the peptide level, also unveiling protein modifications as PTMs or single nucleotide polymorphisms and implying no absence of restrictions to the organism of sample origin. Also, variations in protein structure, leading to alterations of epitopes may still be identified with high confidence using this MS-based approach, covering one major disadvantage of affinity-based proteomics. The sample throughput of this method remains, however, far behind the possibilities of other automated affinity proteomic workflows and might not be initially feasible for large-scale clinical studies.

## Conclusion and Outlook

The proteomic technologies discussed here all carry advantages and shortcomings. Each of them shines with respect to a specific aspect that may be taken advantage of when used in the right spot of a biomarker/drug target discovery pipeline. Current advances in affinity-based proteomics enable the high-throughput analysis needed for large clinical trials to develop biomarkers and investigate molecular mechanisms of disease and treatment response. The major advantage of high-multiplex platforms such as Olink Explore and SomaScan is their reduced analytical time. MS-based proteomic approaches require extensive prefractionation steps in order to acquire a comparable depth of analysis, severely hampering sample throughput. The increasing proteome coverage of recent affinity-based proteomics platforms as well as their excellent detection of low-abundance proteins makes them highly attractive for discovery studies. Other affinity-based approaches, such as Luminex or SiMoa do not provide the same extended proteome coverage as the flagships from Olink or Somalogic, but offer even higher sensitivity for proteins in the low-abundance range, being able to detect even slight alterations in a small panel of biomarkers. Remarkably, these assay formats are also suitable for routine clinical use and have already received FDA approval for a number of diagnostic assays, providing an alternative to the gold standard ELISA. Besides, emerging assay formats such as NULISA continue to push the limits of detection even further, promising high resolution of protein abundance at low concentrations in the near future. Boundaries are also pushed by coordinated combination of MS technology with affinity enrichment of proteins in established MS workflows. Platforms such as those offered by Seer promote high reproducibility through the application of automated sample processing and are increasingly approaching the standards required for clinical proteomics.

Thus, MS- and affinity-based technologies should not be seen as mutually exclusive approaches but as highly complementary tools. For example, changes in PTM prevalence can, as a rule, not be mapped using affinity proteomic technologies. For the detection of PTMs, associated with different diseases, MS-based proteomics are current state of the art. Nevertheless, while protein microarrays do not offer *de novo* identification of such modifications, the analysis of predefined PTMs is well-suited and often used in RPPA approaches to infer pathway activation by the identification of phosphorylation or glycosylation status of specified target proteins in a large number of samples.

In this review, we focus on technical aspects and applicability of affinity proteomic approaches, using the study of the tumor secretome as an example. Harmonized combination of such platforms and MS-based alternatives, either in terms of orthogonal validation or in terms of the sequential steps leading from discovery to actual utility in clinical settings, hold great potential generalizable across biomedical research as a whole.

## Conflict of interest

The authors declare no competing interests.
